# Social Support at School for Students with Sensory Disabilities

**DOI:** 10.3390/ijerph21081071

**Published:** 2024-08-15

**Authors:** Roberta Fadda, Tiziana Piu, Sara Congiu, Doxa Papakonstantinou, Giulia Motzo, Cristina Sechi, Loredana Lucarelli, Ilaria Tatulli, Maria Luisa Pedditzi, Donatella Rita Petretto, Ana Sofia Freire, Konstantinos Papadopoulos

**Affiliations:** 1 Department of Pedagogy, Psychology, Philosophy, University of Cagliari, 09123 Cagliari, Italy; tiziana.piu@unica.it (T.P.); sara.congiu77@unica.it (S.C.); giuliamotzo@gmail.com (G.M.); cristina.sechi@unica.it (C.S.); llucarelli@unica.it (L.L.); pedditzi@unica.it (M.L.P.); drpetretto@unica.it (D.R.P.); 2 Department of Educational and Social Policy, University of Macedonia, 54636 Thessaloniki, Greece; klerip@uom.edu.gr (D.P.); kpapado@uom.edu.gr (K.P.); 3 Department of Literature, Languages and Cultural Heritage, University of Cagliari, 09123 Cagliari, Italy; ilariatatulli@unica.it; 4 Instituto de Educação, University of Lisboa, 1649-013 Lisboa, Portugal; asraposo@ie.ulisboa.pt

**Keywords:** social support, sensory disabilities, visual impairment, hearing impairment, primary school, secondary school

## Abstract

Social support is the gratification of basic social needs (affection, belonging, esteem or approval, security, identity) through interaction with others. Social support at school allows students to perceive themselves as competent during learning and to enjoy school in general. Little is known about social support at school for students with sensory disabilities. This review aims to synthesize findings from studies examining social support at school for students with hearing and visual impairments. A search of computerized databases was supplemented by a manual search of the bibliographies of the main publications. The synthesis of the literature suggests that all students need adequate support devices in class and properly trained support teachers. However, visually impaired students are more likely to have access to resources compared to those with hearing impairments. Students with visual impairments attending regular schools are more positive about the availability of resources than those with hearing impairments attending special schools. Overall, senior secondary school students indicate higher resource availability than junior secondary school ones. Still, very few studies have investigated social support for students with sensory disabilities. Thus, further research is needed to confirm these results.

## 1. Introduction

Sensory disabilities refer to a condition in which one or more sensory functions, such as vision or hearing, are impaired or do not function as expected. In the present literature review, we focus especially on visual impairments, hearing impairments, and deafblindness.

Visual impairment refers to a broad continuum of loss of visual function. Visual function presents various aspects: visual acuity (the ability to distinguish details), accommodation (the ability to focus), visual field (the area that can be seen), color vision, and adaptability to light [1]. Consequently, there are various types and severities of visual impairment. The World Health Organization (WHO) determines the degree of visual impairment by evaluating an individual’s ability to resolve fine detail (visual acuity) through standardized tests. Therefore, visual acuity between <6/18 and 3/60 after correction in both eyes is defined as low vision, and <3/60 is defined as blindness. It should be noted that most people with visual impairments, including those classified as “blind”, retain some residual sight, which can be enhanced for everyday activities and tasks [1].

Hearing impairment is a broad term encompassing various degrees of hearing loss, ranging from mild hearing loss (hypoacusis) to profound deafness [2,3]. An individual is considered to have hearing loss when she or he cannot hear as well as someone with normal hearing, defined as hearing thresholds of 20 dB or better in both ears. Hearing loss can be mild, moderate, severe, or profound. It can affect one or both ears and leads to difficulties in understanding speech and loud sounds. Hard of hearing refers to people with hearing loss ranging from mild to severe. They typically communicate through spoken language and may benefit from hearing aids, assistive listening devices, cochlear implants, and captioning. Deaf individuals primarily have profound hearing loss, meaning they have very little or no hearing. They often rely on sign language for communication [4].

Deafblindness refers not only to a complete absence of hearing and sight but also varying degrees of vision and hearing loss [5]. Even a slight impairment of both vision and hearing can significantly impact daily life. Indeed, the fact that individuals with residual hearing and vision are categorized as deafblind can be attributed to the negative impact of dual sensory loss on the person, where hearing cannot compensate for poor vision and vision cannot compensate for hearing impairment in communication and social interaction [6].

Hearing impairment, visual impairment, or a combination of hearing loss and visual impairment can significantly hamper interaction and learning. Social support is of great importance for students with sensory disabilities to help them face the challenges that can hinder their learning process. Social support at school is a key component of students’ well-being and a critical determinant of public health. It affects mental, emotional, and physical well-being, influences behavior, enhances academic success, and fosters social development. By creating supportive and inclusive environments, schools can significantly contribute to the health and well-being of their students, with long-lasting effects on public health.

### 1.1. Social Support

Social support begins in utero and is communicated in different ways, especially by the way the baby is held (supported) by his or her mother. As the baby grows up, support is provided by other family members, and then by co-workers and the community or members of helping professions in the event of special needs. Towards the end of life, social support is provided mostly by family members [7]. Social support is a complex and multidimensional construct; indeed, several definitions that emphasize different aspects have been proposed over the years. Social support has been defined as those “resources provided by other persons” [8] that lead an individual “to believe that he is cared for and loved, esteemed, valued, and that he belongs to a network of communication and mutual obligation” [7]. Social support is also defined as the gratification of a person’s basic social needs (affection, belonging, esteem or approval, security, and identity) through interaction with others [9]. In general, according to [10], social support refers to “the type of assistance that individuals receive or expect to receive from those who come into contact with them in any way”. The latter definition allows us to highlight the importance of two subconstructs of social support, namely received support (“what individuals receive”) and perceived support (“what individuals expect to receive”).

Received support refers to helping behaviors provided to individuals by members of their social network [11].

Perceived support refers to the belief that helping behaviors are provided when needed [12].

Essentially, received support regards helping behaviors that did happen, whereas perceived support regards helping behaviors that might occur [13].

Social support can be divided into two main types.

Practical social support (or instrumental support) involves the provision of advice and information as well as tangible assistance, such as physical devices, transportation [14], services, and materials [15].

Emotional social support refers to demonstrations of caring, love, empathy, sympathy, understanding, and esteem from significant others [7,9,16,17].

Social support originates from the interconnection between three different types of support resources.

The formal support system includes assistance provided by members of public or private organizations [18].

The informal support system refers to assistance provided by family, friends, and neighbors [19,20].

The quasi-formal support system includes assistance provided by the community (e.g., church-related support groups and activities) [21].

Social support is typically measured through its structural and functional properties.

Structural properties include the size, density, frequency of contact, kinship reliance, accessibility, and stability of an individual’s social network [22,23,24].

Functional properties refer to the amount and adequacy of the specific functions (practical and emotional) provided by social network members [25].

The structure of the social network defines the type of functional assistance to which individuals are exposed [26]. Indeed, the structure’s size and the types of relationships influence the type of received social support [13,27]; received social support, in turn, promotes the perception of the availability of social support [28].

Social support certainly helps individuals by making them feel concretely supported and cared for; however, we will see that the effects are not always positive. Indeed, social support itself may have negative effects on the recipient if it is not requested or, in general, if it does not match what he or she needs.

### 1.2. Positive Social Support and Negative Social Support

It has been shown that some individuals who experience many negative events in their life do not become much distressed, whereas others who go through very few negative events can become highly distressed [29]. To help explain this, researchers have found that persons protected from bad consequences originating from negative events are those who have a network of helpers. Indeed, positive social support has beneficial effects on mental and physical health both in stressful and non-stressful conditions, and it buffers the negative effects of certain events [30]. In particular, it has been shown that emotional support is the most beneficial type of support and the most powerful predictor of reduced psychological distress [16]. Indeed, positive emotional support can increase the recipient’s sense of competence, self-worth, and social connection, and it can also help the subject to reorganize the experience to make it less stressful. Furthermore, it can distract the recipient from concerns regarding the stressful experience, and it can also help him or her to relax physically and promote the use of various coping strategies [31]. Specifically, it has been shown that positive effects can be obtained mostly with “invisible support”, i.e., when individuals do not know they are receiving help. When they are aware of it, benefits are canceled [32]. What applies to emotional support also applies to practical support. On one hand, positive practical support helps individuals have the strength to successfully cope with stressful situations, and it can provide them with more time to relax and sleep; on the other hand, when recipients are aware of receiving practical support, benefits that would derive from it are canceled out [32].

As a result, social support is not always positive, and we can indeed have negative social support. In this respect, practical support can undermine one’s sense of competence and autonomy, and it can prompt an individual to focus on the stressful problem and make him or her feel in debt towards others [32]. In general, negative social support can occur through inappropriate and unnecessary help; accordingly, it can make the recipient feel overprotected [15,33] and believe that his or her own abilities are being underestimated or overestimated [10].

For social support to be effective, a high degree of “fit” between what an individual needs and what is offered is required. Achieving such a “fit” can be facilitated through empathetic understanding based on social and experiential similarities with the person in need [16]. The results of the studies described so far concern studies conducted on adults in the workplace; however, social support can also be important for well-being at school.

### 1.3. Social Support at School for Students with Sensory Disabilities

Social support at school allows students to perceive themselves as competent individuals during the learning process and to enjoy school in general. In the school environment, social support is provided by teachers and classmates [34].

The most important role is played especially by teachers, probably because they are adults and the official leaders of student groups, thus representing a powerful model of behavior.

Teachers have the power to ensure that students feel highly esteemed and valuable by creating a sensitive environment, preventing bullying, and dealing constructively with conflicts [34]. Therefore, students who perceive that their teachers are available to help with academic and social problems [35], care about them [36], and provide positive feedback and clear structure [37] can develop their diverse capacities to achieve the desired results, and they are more motivated in general [38].

Similarly, students tend to be more emotionally, socially, and academically engaged with school if they feel accepted by classmates [39], perceive that classmates care about them [40], and help each other (e.g., helping each other with interpreting and clarifying teachers’ instructions) [38].

In contrast, if students feel rejected and not supported by classmates, they are at risk of behavioral problems and lower interest and participation in school [41].

Social support thus provided by both teachers and classmates is necessary to address the needs of students with disabilities as well. The role of teachers is crucial for students with disabilities as they have the power to create a positive, safe, and inclusive environment that celebrates diversity in which every student has a place and a voice; hence, students with disabilities can enjoy the opportunity to develop positive relationships with classmates, and they can also feel valued and safe, with a strong sense of belonging to the classroom [42]. However, little is known about social support at school for students with sensory disabilities.

The present literature review focuses on social support for students with sensory disabilities, especially visual impairment, hearing impairment, and deafblindness.

The assessment of visual function in the school context is of great importance, as sight plays a crucial role in relating the different types of sensory information during the learning and development process; sight integrates and coordinates the information received through the other senses. Thus, visual impairment reduces the quantity and quality of information available to the student [7]. Indeed, unlike peers who do not have a visual impairment, children with such a disability have limited opportunities to explore the environment, to learn through random and unplanned experiences, and to refine motor skills through observation and imitation of actions performed by others [43]. Providing the necessary supports allows students with visual impairment to adequately perform the same activities carried out by their classmates and promotes social inclusion. In particular, support provided by teachers is crucial. Indeed, teachers must be highly qualified in order to have the necessary knowledge to use all of the electronic devices needed by the students and to support them in their learning process.

The main challenge that students with hearing impairment deal with concerns communicative skills; indeed, such skills are influenced by personality, intelligence, the nature and severity of the hearing impairment, the extent of the benefit derived from hearing aids, the family environment, and the age of onset of the disability [44,45]. Also, concerning students with hearing impairments, the role of the teachers in supporting their development and learning process is crucial; by providing the necessary support (e.g., learning to use all of the necessary electronic devices and learning sign language), their educational process will be facilitated and their quality of life improved.

In summary, social support at school is of great importance for students with sensory disabilities. Meeting their needs can significantly impact their motivation, academic success, and overall well-being within the school environment. The support provided by teachers and classmates enables these students to participate fully in classroom activities and to access a wider range of opportunities, thereby mitigating the challenges caused by their specific disability. In this way, social support creates a safer and more inclusive environment, and it improves the accessibility of the learning process.

The purpose of this review was to synthesize findings about social support for students with sensory disabilities during and before the COVID-19 pandemic. Our review addresses the following questions. (1) Which specific challenges linked to social support at school for students with sensory disabilities (visual impairment or hearing impairments) emerged during the COVID-19 pandemic? (2) What is known about the types of social support received by students with a specific sensory disability (visual impairment vs. hearing impairments) or both (a combination of visual and hearing impairments)? (3) Whether social support changes with age.

## 2. Materials and Methods

This is a narrative review of the studies examining social support at school for students with hearing and visual impairments.

### 2.1. Search Strategies

An extensive literature search was performed by incorporating different search strategies. Initially, the databases PubMed, Scopus, and Isi Web of Knowledge were reviewed. For the purposes of this review, we used different related terms in order to increase the sensitivity of the search. Three terms, “social support”, “hearing impairment”, and “visual impairment”, were considered fundamental in combination with the term “students”. Our search included the terms [“social support” AND “visual impairment” OR “hearing impairment” AND “students”]. The COVID-19 pandemic has presented students with numerous challenges that could represent a source of stress. Therefore, we included the COVID-19 pandemic in combination with the other terms. Thereafter, duplicates were excluded and reference lists from the collected articles as well as from review articles and books on the subject were examined for potentially eligible studies. The searches covered all previously published studies from the first available date until January 2022, when the last search was conducted. Study eligibility and quality were assessed by the authors independently, and the inclusion of studies was based on consensus.

### 2.2. Inclusion Criteria

To be included in this review, studies were required to meet the following inclusion criteria.

First, the studies had to focus on the needs of and social support for students with sensory disabilities in different school grades from kindergarten to senior secondary school. Second, the articles had to be published in English. Studies that did not include students with sensory disabilities were excluded. Qualitative studies, intervention studies, nonhuman animal studies, book chapters, reviews, and studies written in a language other than English were also excluded.

### 2.3. Coding

For the studies that met the inclusion criteria, the following information was coded: (a) groups of participants (students with sensory disabilities); (b) sample size and mean age or educational stage; (c) country of the study; and (d) main objectives and results regarding the needs of and social support for students with sensory disabilities.

## 3. Results

Once duplicates were removed, the search produced 42 records. All of these studies met the inclusion criteria. All of the studies were published in international peer-reviewed journals. By applying the inclusion criteria, 27 records were excluded. We included in our literature review 15 articles, as follows: 6 papers considered the condition of students with sensory disabilities during COVID-19, while 9 papers considered social support at school before the pandemic.

### 3.1. Students with Sensory Disabilities during COVID-19

In December 2019, an outbreak of Coronavirus disease 2019 (COVID-19), a highly contagious respiratory disease, occurred in Wuhan, China. In a few months, the virus spread worldwide, and on 11 March 2020, the World Health Organization (WHO) declared the novel coronavirus outbreak a pandemic. Governments soon established measures in order to prevent the transmission of COVID-19, such as social distancing, wearing face masks, and frequent hand-washing, along with identifying, isolating, and treating active cases [46]. Due to the global emergency, many countries instituted a nationwide lockdown to reduce the risk of contagion; schools, colleges, offices, and other institutions were closed [47]. The restriction of the population’s movement, the fear of being infected, and the unpredictable pandemic trends inevitably influenced the everyday and professional lives of all people; physical contact and communication between people were compromised [48], leading to psychological distress. For instance, in the UK, mental health problems significantly increased by over 50% during the first lockdown [49]. Furthermore, sleep disorders are considered an early health indicator of the general population [46]; in Italy, sleep disorders were one of the main health problems and increased by 57.1% during the first lockdown [50].

The restriction of the population’s movement and the closure of institutions led people to reorganize their activities. For instance, schools and colleges shifted to online classrooms so that students did not experience interruptions in their learning process. Shifting to digital platforms influenced students’ and teachers’ lives and the overall quality of education. In some cases, schools managed to cope with the transition to online platforms, while in other cases numerous difficulties were encountered due to the lack of technological infrastructure [47]. Students became more distracted while attending lessons at home and, as a result, their motivation to learn decreased, negatively affecting their mental health through adjustment, coping, and stress issues. Teachers were also affected by the closure of educational systems as they encountered numerous difficulties in using online platforms due to a lack of technological knowledge and, in some cases, they did not have the means to organize online lessons. This experience impacted their mental health, leading to increased stress and anxiety.

Social support for students with sensory disabilities in the school context was severely hindered by the COVID-19 pandemic. The introduction of new protocols had a negative impact, especially for students with disabilities. Distance learning was developed for pupils without special needs. Students with visual and hearing impairments encountered numerous difficulties in using online platforms and there were no lessons designed for them [51]. Before the pandemic, students with visual impairments attended specialized schools for the blind or spent part of the time in specialized schools and part of the time in public schools with support. Other students were taught by a specialized teacher at home, in a private school, or in a hospital. Teachers of students with visual impairments would provide them and their teachers with the materials they needed, such as specialized materials and braille or large-print materials, or instruction in the expanded core curriculum [52]. Similarly, intervention programs were usually developed according to the needs of students with hearing impairments. They would participate in a face-to-face therapeutic process with game-based learning methods and participate in recreational and educational activities with logopedic methodologies in order to work on all of the components of language. At the same time, therapists used to offer guidance to parents and caregivers, who would actively participate [53]. Since the global COVID-19 pandemic, such methods were no longer possible as a shift from in-person to remote or virtual modalities occurred. The special education teachers available before the pandemic were no longer available for most students with visual and hearing impairments. Furthermore, relationships with classmates were significantly compromised, worsening considerably [46].

Teachers of students with visual impairments often had to go through a process of trial and error with their students in order to figure out how to turn audio and video on while using the Zoom platform for remote lessons. Furthermore, they had to develop workarounds by collaborating with general education teachers in order to allow blind pupils to access chats and online quizzes, as mainstream platforms used by schools were designed for sighted peers. Nevertheless, positive outcomes were achieved, as teachers were prompt to be creative in order to teach and involve students with visual impairments during online lessons. Moreover, the involvement of parents in their children’s education increased, thus improving communication and relationships between teachers and their students’ families [54].

Students with hearing impairments encountered technical issues during online lessons: their hearing devices did not always manage to accurately capture speech or sounds, thus making it difficult for them to understand the transmitted material. Most importantly, there were no transcription or subtitles provided while the teacher was speaking. This poses a significant issue, especially for students who rely heavily on lip reading. All of these challenges result in a significant impact on the emotional dimension, thereby contributing to emotional stress [55]. Furthermore, communication between teachers of students with hearing impairments and their parents worsened, thus affecting parent–teacher collaboration. When school reopened, students with hearing impairments had to deal with the one-meter social distancing rule and the use of masks, which hindered lip reading and muffled the teacher’s voice [51]. Thus, teachers should be prepared for unexpected service model changes, ensuring access to materials and technology for visually impaired students at home and at school. Recommendations include instructing families, implementing backup systems, and monitoring student progress during crises [52]. For deaf or hearing-impaired students, strategies involve frequency modulation hearing systems and early reading skill promotion. Further research on developing communication skills through online role-play is proposed [55]. In summary, the pandemic has been an extreme situation that significantly hindered the possibility of receiving adequate social support for students with sensory disabilities. For this reason, the good practices applied before the pandemic should be known and enriched at different school ages.

### 3.2. Research Concerning Social Support for Students with Sensory Disabilities

Nine studies investigating different perspectives of social support at school for students with sensory disabilities were selected. We decided to consider them according to the types of sensory disabilities of the students involved in the studies. This criterion will allow us to highlight the similarities and differences that characterize the kind of social support that is necessary for students with different sensory disabilities. This understanding will be crucial to develop effective intervention studies to promote social support at school, both from teachers and peers. According to this criterion, we will discuss the main results of two studies focusing on deafblind students [56,57], three studies focusing on deaf/hard-of-hearing students [58,59,60], and, finally, four studies focusing on both students with visual impairments and students with hearing impairments [61,62,63,64].

#### 3.2.1. Studies on Deafblind Students

The study conducted by Grell and Howard (1981) [56] involved 17 deafblind students from the Iowa School for the Deaf; the largest percentage (53%) of these students fell between the ages of 14 and 16. Their vision loss was largely caused by Rubella and Usher’s Syndrome. The study was aimed at implementing a deafblind resource room in order to meet the individual needs (depending on their different ages, the severity of visual and hearing loss, the prognosis, and academic abilities) of deafblind students. They attended the resource room for as many as three classes per day in order to receive suitable and additional supports beyond those they had in the deaf classroom, such as using the typewriter, braille, and canes in mobility training, all provided by a certified teacher. The objectives of the resource room were, for each student, the following:

1. To develop adaptation skills specific to the visual–auditory handicapping condition;
2. To provide tutorial help to students in instructional areas;
3. To develop students’ social and psychological adequacy;
4. To assist students in developing survival skills for life.

Regarding that study, the best evaluation of the resource room would have occurred within 5–10 years after its publication, when most of the Rubella and Usher’s Syndrome population entered the workforce. In the meantime, indicators of success looked promising. Indeed, continuous improvement by the program was found in the areas of educational assessment and evaluation, medical diagnosis and evaluation, implementing an Individual Educational Plan (IEP), and providing various services. Nonetheless, the area of family services encountered some issues, as it was difficult to schedule continuous meetings with parents due to the distance of most homes from the school.

Goetz and O’Farrell (1999) [57] in their study involved four deafblind students (a 10-year-old student, a fifth-grader, and two kindergartners) in general education classrooms in northern California. The paper presented Connections, a three-year project aiming to use Hunt, Alwell, Farron-Davis, and Goetz’s [65] three-component intervention model in order to facilitate social support for deafblind students and promote their participation as valued members of their general education classrooms. The social support package included three strategies, as follows:

1. The ongoing provision of information about the individual student’s needs and strengths and about deafblindness through various activities and formats in the classroom [66].
2. The provision of interactive, multimedia communication systems that are appropriate for the individual student’s needs and in classroom contexts [67].
3. The continuous facilitation of social interactions among classmates through various activities and strategies [68].

The results showed that for the four students, active engagement in the different activities of the general education classroom occurred during at least 70% of their school day; students with “typical” cognitive abilities had no more interactions with their classmates than those with cognitive impairments; and interactions classified as “assists” by the staff also did not increase according to the severity of the disability.

Table 1 illustrates the main objectives and results of the studies on deafblind students.

In general, there is a beneficial effect of making accessible the devices to support the students with disabilities. However, support for the families is a criticism very difficult to overcome. Facilitating social interaction in mainstream classrooms improves active engagement by students with disabilities, regardless of their cognitive functioning. Thus, it is worth implementing this kind of intervention at school at different ages. However, as it is possible to see, the studies considering students with a combination of deafness and blindness are rare. Moreover, the two studies considered small samples with heterogeneous ages. Also, the schools adopted different protocols to promote social support at school. Thus, it is difficult to compare the results and to drive specific guidelines for successful interventions at school.

#### 3.2.2. Studies on Deaf/Hard-of-Hearing Students

The study carried out by Angelides and Aravi (2007) [58] took place in a secondary school in Cyprus, with students aged 13–15. In particular, the study focused on two classes with deaf/hard-of-hearing (D/HH) students: one for first-graders, with four D/HH students, and one for second-graders, with three D/HH students. The main purpose of the study was to analyze the impact that the integration of D/HH students had on the development of inclusive practices and on the development of the school in general. The results indicated that the presence of D/HH pupils stimulated teachers to differentiate their teaching by simplifying the texts of their lessons in order to be comprehended by D/HH students and to introduce innovation, such as the use of an overhead projector. The integration of D/HH pupils also stimulated teachers to develop collaborations among them and to exchange ideas and practices in order to make their working methods more effective. Finally, the integration of D/HH students prompted teachers to develop more inclusive practices that they implemented in classes for all of the children with great success. Thus, the integration of D/HH pupils had a positive impact on the development of inclusive practices and of the school in general, as organizational and methodological changes benefited not only D/HH students but also all other students.

The study accomplished by Kelman and Branco (2009) [59] focused on four bilingual classes for the deaf (each with a coteaching team formed by a regular teacher interacting with a special educator who uses sign language) in three public elementary schools in Brasília, Brazil. Each classroom had an average of 25 students, with 6 being deaf. The aim of the study was to identify the elements of success for an inclusive classroom. In particular, the authors focused on communicative and metacommunicative strategies, identifying those that would promote or hinder the process of inclusion of deaf students in such classes. By analyzing social interactions in the classrooms through a microgenetic method based on naturalistic observation and video-taped sessions, inclusive episodes and non-inclusive episodes were selected. The results showed different inclusive episodes. During an explanation, a regular teacher showed tenderness to her deaf students through body postures and facial expressions, such as smiles, touches, and bending on her knees to get closer to them. A regular teacher was writing the correct answers to some problems on the board, and a deaf student warned her that she had written a wrong answer, suggesting that the class was so integrative that the student felt comfortable correcting the teacher. A specialized teacher taught sign language to the whole class, fostering communication between hearing students and their deaf peers, therefore promoting inclusion. Hearing pupils discovered the situations in which sign language was useful and they used it for distance communication, therefore promoting interest in becoming bilingual. The results also showed different non-inclusive episodes. A deaf student asked the specialized teacher to ask a hearing student for a sharpener, suggesting that the student did not feel comfortable asking his classmate on his own, and the teacher, acting as an intermediary in the process, inhibited interaction between hearing and deaf students, thus discouraging inclusion. A specialized teacher ignored the deaf pupil’s requests for attention, generating frustration, humiliation, and a feeling of insecurity. A deaf student gently touched the earrings of the specialized teacher who rejected her, generating aggressiveness in the student and refusal to complete the math exercise she was doing, thus negatively impacting the performance of the academic task.

The study accomplished by Cheng, Deng, and Yang (2020) [60] involved 225 D/HH students from 6th to 11th grade in mainland China. The main purpose of the study was to investigate the contributions of social support to student engagement, taking demographic variables into consideration. Data were collected by administering the Chinese Version of Zimet’s Multidimensional Scale of Perceived Social Support (MSPSS-C) and the Student Engagement Scale. The results demonstrated that D/HH students with higher levels of social support scored higher on student engagement, whereas those with lower levels of social support had lower scores.

A summary of the studies will be presented below (Table 2), indicating the main objectives and results of the studies on deaf and hard-of-hearing students.

As it is possible to see, there is a general positive effect of including deaf/hard-of-hearing students in mainstream classes. Teachers are more likely to adopt effective and inclusive teaching practices, with a beneficial effect for all students. However, the results are not straightforward, as non-inclusive interactions have been documented, especially among peers. This means that interventions should focus not only on training teachers but also classmates to teach them how to recognize the needs of deaf/hard-of-hearing students and how to offer them effective social support at school. This is particularly worthwhile because it seems that deaf/hard-of-hearing students who receive social support tend to score higher on student engagement in the activities of the class.

#### 3.2.3. Studies on Students with Visual Impairments and Students with Hearing Impairments

The study conducted by Byrne (2014) [61] included 20 students with visual and hearing impairments in third-level education. The study had two aims: firstly, to analyze the experiences reported by these students in accessing and engaging with support provisions; and secondly, to identify the different ways in which these students managed the practices they encountered. The results showed that some students were impressed by the level of support provided, whereas other students had negative experiences due to a lack of support. Moreover, some education providers argued that the lack of complaints was a positive indicator of good practice; in fact, some students did not feel comfortable externalizing their needs. Furthermore, in some cases, the students had to request the supports they needed. Some of them had no problem with this practice, while others felt that waiting in order to obtain the necessary supports hindered the learning process. Regarding specifically students with visual impairments, some of them reported many difficulties as the further education courses they enrolled in provided support that was largely suitable for students with learning disabilities. For this reason, one of these students asked to be moved to another course. Other students reported dealing with inadequately trained support workers, resulting in the students adapting to their level. With respect to students with hearing impairments, according to some of them, the support had been more of an obstacle than a help. Indeed, they had to take care of paying their assistants, organizing meetings, and filling out lots of papers and forms. Also concerning students with hearing impairments, they had to deal with inadequately trained interpreters and therefore had to adapt to the situation. Finally, some of them were forced to stay with their interpreters in front of all of the other students, thus feeling embarrassed; once the digital hearing aids were received, the problem was solved. On the other hand, other students with digital hearing aids did not feel comfortable wearing them, trying therefore to hide them (e.g., wearing their hair down). In conclusion, the study highlighted that disability advisers and support provisions are good practices that were beginning to emerge in third-level settings; however, third-level education seemed to be characterized by complex forms and practices of discrimination.

Genç and Koçdar (2020) [62], in the first phase of their study, involved 18 volunteer learners with hearing impairments, visual impairments, and physical disabilities enrolled in the Open Education System of Anadolu University, with 6 in each group. The second phase included 703 volunteer learners with these three types of disability enrolled in the Open Education System. The main purpose of the study was to identify the needs and priorities of learners with special needs for support services in an open and distance teaching university in Turkey based on disability type. Support service needs were identified in the first phase via semi-structured interviews; support service priorities were identified in the second phase through three surveys developed specifically for each disability type, Determining the Priorities of Learners with Special Needs in Support Services (DPLSNSS), which were sent via e-mail and SMS. In particular, data were collected regarding managerial, educational, social, technical, and vocational support. We report below the needs and priorities for support services of learners with visual and hearing impairments that we are addressing. In the context of managerial support, learners with hearing impairments required support for basic needs, such as using the restroom, etc., in the waiting period before the on-site exam and rest breaks during the exam. Students with visual impairments needed to register independently and be supported in meeting relatives after the exams, and they needed the accessibility of the university buildings to improve. As regards priorities for managerial support services, both participants with hearing and visual impairments designated the items “remind learners about registration and examination dates via SMS” and “inform learners about the services offered by Open Education System via SMS” as most important and “allow rest breaks during the exam if it is necessary” as least important. In the context of educational support, students with hearing impairments needed simplified books, sign language interpreters for video content, and more access to library services; as regards priorities, they designated the item “provide captioning for webinars” as most important and “assign an academic supervisor for each learner with special needs” as least important. Learners with visual impairments also required simplified books and audio books vocalized by experts and with natural sound, and they needed learning materials to be compatible with screen readers. Also, they prioritized Word format for learning materials while they found Braille format least important. In the context of social support, both participants with hearing and visual impairments needed social activities to be organized, and they also needed psychological support to be offered. Furthermore, they both prioritized “conduct responsibility projects for the learners with special needs under the leadership of the university”. In the context of technical support, both learners with hearing and visual impairments needed support in using the website of the university. Regarding priorities for technical support services, participants with hearing impairment designated the item “offer easy access to e-campus from university website” as most important, whereas students with visual impairment designated the items “offer easy access to e-campus from university website” and “increase the usability of e-campus website” as most important. Finally, in the context of vocational support, students with hearing impairments needed vocational guidance, and they designated the item “include links to job announcements for the learners with special needs on the university website” as most important. On the other hand, participants with visual impairment designated the item “include links to internship announcements for the learners with special needs on the university website” as the most important.

The study accomplished by Kisanga (2020) [63] involved 27 students with visual and hearing impairments in Tanzania’s higher-educational institutions. The study had the following three aims: to analyze what coping strategies the students with sensory impairments (SIs) would employ to overcome the academic barriers they faced in their studies; to analyze what coping strategies they would use to overcome the social barriers they faced in their studies; and to analyze how the coping strategies of students with SIs would differ according to their demographic characteristics. Data were collected via focus group discussions, semi-structured interviews, and an open-ended questionnaire. The results indicated that students with SIs employed problem-focused strategies more than emotion-focused coping to overcome the academic barriers, namely support networks, their own efforts, and collective efforts. The analysis of the second research question indicated that students with SIs utilized emotion-focused strategies more than problem-focused coping to overcome social barriers. Thus, they sought to change their negative feelings into positive ones in order to cope. Students with SIs employed both positive emotion-focused strategies, such as accepting their impairment and perceiving their impairments as an opportunity rather than a problem, and negative emotion-focused strategies, such as distancing from the problems they face. Regarding the last research question, the analysis of data revealed differences between male and female students with visual impairments (VIs) in the coping strategy selected: males made an effort by creating a mental picture of an area, whereas female students depended on social support networks to move around. Differences were also found in the coping strategies employed by students with visual impairments between the lower and higher levels of education. In primary school, they depended more on their parents; in ordinary-level secondary school, they depended on their peers; and in advanced-level secondary school, they were able to use computers with assistive technology to access educational materials in electronic format.

The study conducted by Opoku, Nketsia, Fianyi (2020) [64] recruited 115 students with sensory disabilities from one special and two regular secondary schools in Ghana. The main purpose of the study was to analyze the perspectives of students with sensory disabilities on resource availability. More specifically, the study aimed to examine the association between the profile of these students and the availability of resources, to analyze the interaction effect of these students with sensory disabilities on other demographic variables and the availability of resources, and, finally, to identify the variables that would influence the likelihood of these students accessing resources. Data were collected through a questionnaire evaluating the demographic variables in the first section and the availability of resources in the second section via the Perceived Resources Questionnaires (PRQs). With respect to the first objective, the results revealed differences concerning disability type, gender, and study grade. The visually impaired students scored higher on resource availability than their hearing-impaired counterparts. Moreover, students with visual impairments attending regular schools were more positive about the availability of resources than those with hearing impairments attending special schools; the male participants were more positive about resource availability than the female students; and students in the early years of study were more positive about the perceived availability of resources than those in later grades. As regards the second objective, an interesting variable was the interaction between disability type and educational level. Overall, the students attending senior secondary school indicated higher resource availability than those in junior secondary school. However, hearing-impaired students attending senior secondary school were more positive than those attending junior secondary school, whereas visually impaired students in junior secondary schools indicated more resource support than those attending senior secondary schools. Finally, regarding the last objective, the strongest predictor was students’ grades: students with visual impairments in higher grades were more than twice as likely to have access to resources than those in lower grades. Moreover, students with visual impairments in junior secondary school were more likely to have access to resources than those in senior secondary school. Finally, students with visual impairments were more likely to have access to resources compared to those with hearing impairments.

A summary of the four studies will be presented in Table 3, indicating the main objectives and results. All of the studies considered older students, with a particular focus on students at the university level and in secondary school. This means that research about pupils in primary schools and middle schools is missing, highlighting the importance of focusing on younger ages in future studies. Regarding the need for social support, it seems that there are still several social barriers both at the university level and in secondary schools. To overcome these barriers, students require, in general, more social opportunities to spend time with their peers and more access to information about relevant opportunities for their lives, like, for example, job opportunities. However, as it is possible to see, there are significant cultural differences running across the studies, with two of them conducted in least-developed countries, Ghana and Tanzania. The studies point to the need to consider cultural and socio-economic dimensions as possible mediating variables influencing the quality of life at school and in university of students with sensory disabilities.

## 4. Discussion

The main purpose of this systematic review was to synthesize findings from studies examining social support at school for students with hearing and visual impairments. We aimed to answer three research questions. (1) Which specific challenges linked to social support at school for students with sensory disabilities (visual impairment or hearing impairments) emerged during the COVID-19 pandemic? (2) What is known about the types of social support received by students with a specific sensory disability (visual impairment vs. hearing impairments) or both (a combination of visual and hearing impairments)? (3) Whether social support changes with age.

Regarding the first research question, students with visual and hearing impairments encountered numerous difficulties during the pandemic, especially in using online platforms. At the same time, this prompted some teachers to be creative and to find effective ways to communicate with them. However, the relationships with classmates were significantly compromised. Furthermore, the communication between teachers and parents worsened considerably, thus negatively affecting parent–teacher collaboration. All of these challenges contributed to increased emotional stress in the students with sensory disability, pointing to the importance of highlighting and enriching the best practices that have been used before the pandemic.

In this direction, the results of our review suggested that all of the students need adequate support devices in order to follow the lessons at school and properly trained support teachers. The differences in social support in these two groups of students are evident in the availability of resources at school. Indeed, visually impaired students scored higher on resource availability than their hearing-impaired counterparts; they also were more likely to have access to resources compared to those with hearing impairments. Finally, students with visual impairments attending regular schools were more positive about the availability of resources than those with hearing impairments attending special schools. These results indicate that students with hearing impairments receive less social support at school compared to visually impaired students. Indeed, students with hearing impairments can succeed in regular schools where deaf culture is respected and sign language interpretation services are available, facilitating communication between them and teachers [69]. However, these results concern countries where sign language is used exclusively in special schools for students with hearing impairments. Therefore, in mainstream schools, there is a lack of specific supports and special educators, making it difficult for these students to participate in regular classes and thus leading them to attend special schools where the necessary supports are provided.

Regarding the third research question, we found that, overall, the students attending senior secondary school indicated higher resource availability than those in junior secondary school. Nonetheless, hearing-impaired students attending senior secondary school were more positive than those attending junior secondary school, while visually impaired students in junior secondary schools indicated more resource support than those attending senior secondary schools. The results confirm that the condition of students with hearing and visual impairments changes with age in a specific way. Again, the findings can be attributed to the specific policies of the countries where the studies were conducted. In these regions, a recent emphasis on equitable upper-secondary education has resulted in free tuition and increased resource allocation. Consequently, students with sensory disabilities attending upper secondary schools have benefited from greater resource availability compared to those in lower-secondary schools. However, the difference in outcomes across different disabilities and educational levels requires further investigation.

The findings of this research may help policymakers to understand better the experiences of students with sensory disabilities at school. Thus, it is necessary to develop individualized interventions to address the specific needs of each population considered and to allocate more resources to schools to facilitate inclusive practices. This would enable both visually and hearing-impaired students to participate fully in regular classrooms. Additionally, policymakers should consider the diverse needs of students with sensory disabilities across different age groups when designing interventions.

However, considering the paucity of studies conducted so far about social support at school for students with sensory disabilities, more studies are needed to confirm these results. This work has some limitations that need to be acknowledged. First, the selected studies were conducted in several geographical locations (Iowa, California, Cyprus, Brazil, China, Turkey, Tanzania, and Ghana). Indeed, the diversity of cultures might be reflected in the schools’ organization. For this reason, the results of the study might not be generalizable across different countries and different cultures. Second, some of the studies considered very small samples of subjects. Again, this might render the results hard to generalize to a broader population of students. Finally, the selected studies involved students of rather different ages, from kindergarten to university. Thus, the studies used heterogeneous social support assessment tools to account for the characteristics of the samples considered. This might render it very difficult to compare the results between the different studies. Future research should include an analysis of social support needs across other disabilities to investigate similarities and differences compared to sensory disabilities.

## 5. Conclusions

Social support at school plays a crucial role in promoting public health, contributing to the well-being and development of students with sensory disabilities. Social support from peers, teachers, and school staff can improve students’ well-being. Positive relationships and a supportive environment can foster a sense of belonging and self-worth. Thus, when students feel supported, they are more likely to engage in learning, participate in class, and seek help when needed. At the same time, supportive interactions help students develop communication, empathy, conflict resolution, and problem-solving skills. Despite the central role of social support at school for students’ well-being and development, little is known about social support at school for students with sensory disabilities. This review synthesized findings from studies examining social support at school for students with hearing and visual impairments. Remarkably, we found that the COVID-19 pandemic hampered the possibility for teachers and classmates to support students with sensory disabilities. This points to the importance to giving voice to their needs and to highlight the best practices applied at school before the pandemic. The studies that we considered in our literature review indicated that students with sensory disabilities mainly need to use adequate support devices in class and require properly trained support teachers. However, visually impaired students were more likely to have access to resources compared to those with hearing impairments. Also, students with visual impairments were more positive about the availability of resources than those with hearing impairments. Overall, senior secondary school students indicated higher resource availability than junior secondary school ones. These results indicated that even though, in general, students with sensory disabilities share the same wants and needs, there are some specific needs that teachers should consider to offer them the best support at school. Also, younger students seem to be more at risk of receiving less support in terms of available resources compared to older ones. Thus, junior secondary school should be a specific target for future interventions to promote social support at school for students with disabilities.

## Figures and Tables

**Table 1:** Studies on deafblind students.

Study	Participants	Objectives	Results
Grell and Howard, 1981 [56]	17 deafblind students. Age: from 8 to 10 to 17 to 19.	To implement a resource room in order to meet the individual needs of deafblind students at the Iowa School for the Deaf.	Continuous improvement in the program in the areas of educational assessment and evaluation, medical diagnosis and evaluation, implementing Individual Educational Plans (IEPs), and providing various services. Difficulty in providing the expected services to families.
Goetz and O’Farrell, 1999 [57]	4 deafblind students. Age: a 10-year-old student, a fifth-grader, and two kindergartners.	To use a three-component package to facilitate social supports for deafblind students and promote their participation as valued members of their general education classrooms in northern California.	For the four students, active engagement in the activities of the general education classroom occurred during at least 70% of their school day. Students with “typical” cognitive abilities had no more interactions with their classmates than those with cognitive impairments. Interactions classified as “assists” by the staff did not increase in relation to the severity of the disability.

**Table 2:** Studies on deaf/hard-of-hearing students.

Study	Participants	Objectives	Results
Angelides and Aravi, 2007 [58]	A class for 1st-graders with 4 D/HH students, and a class for 2nd-graders with 3 D/HH students, in Cyprus. Age: 13–15.	To analyze the impact that the integration of D/HH students had on the development of inclusive practices and on the development of the school in general.	Teachers were stimulated to differentiate their teaching and to introduce innovation; to develop collaborations among them; and to develop more inclusive practices that they implemented in classes for all of the children with great success.
Kelman and Branco, 2009 [59]	Four elementary school classes in Brazil, with an average of 25 students, with 6 being deaf.	To identify the strategies that would promote or hinder the process of inclusion of deaf students.	Inclusive episodes A regular teacher showed tenderness to her deaf students. A deaf student felt comfortable correcting his teacher. A specialized teacher taught sign language to the whole class. Hearing students used sign language for distance communication. Non-inclusive episodes A specialized teacher acted as an intermediary between a deaf and a hearing student. A specialized teacher ignored the deaf pupil’s requests for attention. A specialized teacher refused physical contact with the deaf student.
Cheng, Deng, and Yang, 2020 [60]	225 D/HH students from 6th to 11th grade in mainland China.	To investigate the contributions of social support to student engagement, taking demographic variables into consideration.	D/HH students with higher levels of social support scored higher on student engagement. D/HH students with lower levels of social support had lower scores.

**Table 3:** Studies on students with visual and hearing impairments.

Study	Participants	Objectives	Results
Byrne, 2014 [61]	20 students with visual and hearing impairments in third-level education.	To analyze the experiences reported by students with visual and hearing impairments in accessing and engaging with support provisions. To identify the different ways in which these students managed the practices they encountered.	Disability advisers and support provisions were beginning to emerge in third-level settings; however, the third-level education seemed to be characterized by complex forms and practices of discrimination. Some students had a positive evaluation of the supports received; others were not comfortable with the practices they faced. Visual impairments Some students had inadequate support, thus asking to be moved from one course to another. Some students had inadequately trained assistants and had to adapt to the situation. Hearing impairments In some cases, the support was hindering as students had to take care of paying their assistants, organizing meetings, and filling out lots of papers and forms. They had inadequately trained assistants and had to adapt to the situation. They felt embarrassed as they had to stay with the interpreter in front of the whole class, thus solving the problem by wearing the digital hearing aids. Students with digital hearing aids did not feel comfortable wearing them, trying therefore to hide them.
Genç and Koçdar, 2020 [62]	First phase: 18 volunteer learners with hearing impairments, visual impairments, and physical disabilities. Second phase: 703 volunteer learners with hearing impairments, visual impairments, and physical disabilities.	To identify the needs and priorities of learners with special needs for support services in an open and distance teaching university in Turkey based on disability type.	Results for learners with visual and hearing impairments. Managerial support Learners with hearing impairments required support for basic needs during the exams. Learners with visual impairments needed to register independently and needed support in meeting relatives after the exams; they also needed the accessibility of the university buildings to improve. Both participants with hearing and visual impairments indicated that “remind learners about registration and examination dates via SMS” and “inform learners about the services offered by Open Education System via SMS” were most important, while “allow rest breaks during the exam if it is necessary” was least important. Educational support Learners with hearing impairments needed simplified books, sign language interpreters for video contents, and more access to library services. “Provide captioning for webinars” was most important, while “assign an academic supervisor for each learner with special needs” was least important. Learners with visual impairments required simplified books, audio books vocalized by experts, and learning materials compatible with screen readers. They prioritized Word format for learning materials, while they found Braille format least important. Social support Both participants with hearing and visual impairments needed social activities to be organized and psychological support to be offered. They both prioritized “conduct responsibility projects for the learners with special needs under the leadership of the university”. Technical support Both learners with hearing and visual impairments needed support in using the website of the university. Participants with hearing impairments identified “Offer easy access to e-campus from university website” as most important. Participants with visual impairment identified “Offer easy access to e-campus from university website” and “increase the usability of e-campus website” as most important. Vocational support Learners with hearing impairment needed vocational guidance. “Include links to job announcements for the learners with special needs on the university website” was selected as most important. Participants with visual impairment identified “Include links to internship announcements for the learners with special needs on the university website” as most important.
Kisanga, 2020 [63]	27 students with visual and hearing impairments in Tanzania’s higher-educational institutions.	To analyze the coping strategies employed by students with SIs to overcome academic barriers. To analyze the coping strategies employed by students with SIs to overcome social barriers. To analyze how the coping strategies differed according to their demographic characteristics.	Academic barriers Students with SIs employed problem-focused strategies. Social barriers Students with SIs employed emotion-focused strategies. Differences in students with VIs Gender: males created a mental picture of an area, whereas female students depended on social support networks to move around. Age/educational level: in primary school, they depended more on their parents; in ordinary-level secondary school, they depended on their peers; and in advanced-level secondary school, they were able to use computers with assistive technology to access educational materials in electronic format.
Opoku, Nketsia, Fianyi, and Laryea, 2020 [64]	115 secondary school students with sensory disabilities in Ghana.	To examine the association between the profile of these students and the availability of resources. To analyze the interaction effect of these students with sensory disabilities on other demographic variables and the availability of resources. To identify the variables that influence the likelihood that these students have access to the resources.	First aim Visually impaired students scored higher on resource availability than their hearing-impaired counterparts. Visually impaired students attending regular schools were more positive about the availability of resources than those with hearing impairments attending special schools. Male participants were more positive about resource availability than the female students. Students in the early years of study were more positive about the perceived availability of resources than those in later grades. Second aim Overall, the students attending senior secondary school indicated higher resource availability than those in junior secondary school. Hearing-impaired students attending senior secondary school were more positive than those attending junior secondary school. Visually impaired students in junior secondary schools indicated more resource support than those attending senior secondary schools. Third aim Students with visual impairments in higher grades were more than twice as likely to have access to resources than those in lower grades. Visually impaired students in junior secondary school were more likely to have access to resources than those in senior secondary school. Students with visual impairments were more likely to have access to resources compared to those with hearing impairments.
